# Minimally invasive hyperthermic intraperitoneal chemotherapy at the time of interval cytoreductive surgery for ovarian cancer: a case report

**DOI:** 10.1093/jscr/rjag812

**Published:** 2026-09-16

**Authors:** Eseohi Ehimiaghe, Genna Bonfiglio, Christina Hoang, Spyridon A Mastroyannis

**Affiliations:** Division of Gynecologic Oncology, Obstetrics & Gynecology, University of Colorado Anschutz Medical Campus, 12631 East 17th Avenue, Aurora, CO 80045, United States; Comprehensive Cancer Center, University of Colorado Anschutz Medical Campus, 1665 Aurora Ct, Aurora, CO 80045, United States; Department of Obstetrics & Gynecology, University of Colorado Anschutz Medical Campus, 12631 East 17th Avenue, Aurora, CO 80045, United States; University of Colorado Anschutz Medical Campus, 13001 East 17th Place, Aurora, CO 80045, United States; Division of Gynecologic Oncology, Obstetrics & Gynecology, University of Colorado Anschutz Medical Campus, 12631 East 17th Avenue, Aurora, CO 80045, United States; Comprehensive Cancer Center, University of Colorado Anschutz Medical Campus, 1665 Aurora Ct, Aurora, CO 80045, United States

**Keywords:** epithelial ovarian cancer, HIPEC, minimally invasive surgery, cytoreductive surgery, morbid obesity, financial toxicity, cost efficiency

## Abstract

Ovarian cancer is the second most prevalent gynecologic cancer and the leading cause of death of all gynecologic malignancies. At the time of diagnosis, most patients have advanced-stage disease and neoadjuvant chemotherapy may be used in these patients to increase the chance of optimal cytoreductive surgery (CRS). Hyperthermic intraperitoneal chemotherapy (HIPEC) at the time of interval CRS has been shown to improve both recurrence free and overall survival in eligible patients. We present a case of a patient with a BMI of 54 who underwent a minimally invasive interval cytoreduction followed by HIPEC. This case demonstrates that minimally invasive HIPEC using standard equipment is safe, feasible, and cost effective.

## Introduction

Ovarian cancer is the second most prevalent gynecologic cancer and second leading cause of death of all gynecologic malignancies [1]. In the absence of approaches for early detection, most patients present with advanced stage disease with shorter progression free and overall survival compared to their counterparts with early-stage disease. In 2018 Van Driel *et al*. published Hyperthermic Intraperitoneal Chemotherapy in Ovarian Cancer which showed progression free survival (PFS) and overall survival (OS) with a 3.5 month and 11.8 month benefits with the addition of Hyperthermic Intraperitoneal Chemotherapy (HIPEC), respectively [2]. While there are prognostic benefits to HIPEC, it has also been associated with increased surgical cost. One study estimated a median cost of $32 169 for cytoreductive surgery (CRS) alone compared to $38 405 for CRS with HIPEC. The additional costs are a result of longer surgical times, post-operative hospitalizations, and higher rates of post-operative complications [3, 4]. In recent years, there has been an increase in the use of minimally invasive surgical routes such as robotic and conventional laparoscopy for CRS. A small number of studies have demonstrated safety, shorter length of stay, and acceptable oncologic outcomes for patients who undergo minimally invasive CRS and HIPEC [5, 6]. These studies have performed HIPEC using special laparoscopic equipment that is compatible for the route of surgery. The use of this equipment brings increased costs, with the need for new equipment and additional perfusionist training. In the following case, we describe the use of a standard HIPEC apparatus at the time of a minimally invasive CRS for a patient with stage IIIc epithelial ovarian cancer (EOC) and morbid obesity.

## Case report

A 52 years old patient with a history significant for hypertension and BMI of 54.33 was diagnosed with Stage IIIc EOC after presenting with several weeks of postmenopausal bleeding. Her initial evaluation included a transvaginal ultrasound, which was limited by her habitus, and an endometrial biopsy that was significant for a benign polyp. A computed tomography (CT) of the abdomen/pelvis was performed, which revealed a non-mass-like mesenteric and omental infiltration, and small volume ascites, however, the exam was limited by the patient’s habitus. A diagnostic paracentesis was performed, and cytology confirmed high grade serous adenocarcinoma of mullerian origin. She underwent a PET/CT which showed hypermetabolic activity at and adjacent to the uterus and bilateral adnexa, significant hypermetabolic omental thickening, and mild hypermetabolic retroperitoneal and mediastinal lymph nodes.

In the setting of advanced disease, she was counseled on neoadjuvant chemotherapy followed by interval CRS. She completed 3 cycles of carboplatin and paclitaxel, and underwent a CT, which showed interval disease in the density of peritoneal carcinomatosis. Given the response and findings on CT, the patient was counseled on interval CRS with HIPEC. In the setting of minimal residual disease, she was counseled on a minimally invasive CRS. She was additionally counseled on the benefits of minimally invasive surgery (MIS) for wound healing and post-operative recovery.

## Surgical technique and equipment

The patient was positioned and prepped in the standard manner for MIS gynecologic surgery. Laparoscopic entry and insufflation were performed using direct entry techniques with an 8 mm Airseal at Palmer’s point. Four robotic ports were placed sequentially, one 10 cm left lateral to the umbilicus (robot arm 1), 3–4 cm superior to the umbilicus (arm 2), 8 cm right lateral to the umbilicus (arm 3), and 16 cm right lateral to the umbilicus (arm 4). The DaVinci robotic system was docked. A vessel sealer was placed in arm 1, the camera was placed in arm 2, monopolar scissors were placed in arm 3, and a long prograsp placed in arm 4. A robotic assisted total laparoscopic hysterectomy, bilateral salpingo-oophorectomy, infracolic omentectomy, and pelvic washing were performed. The robot was undocked, the robotic trocars were left in place, and the supraumbilical incision was extended to 4.5 cm in length. The standard laparotomy HIPEC inflow and outflow tubing were introduced through the supraumbilical incision. Hunter graspers were placed in the lateral robotic ports and used to position the appendages of the inflow and outflow tubing throughout the abdomen. Three temperature probes were placed- in the inflow, outflow tubing, and for intra-abdominal temperature monitoring. All the robotic trocars were removed. The 4.5 cm incision was closed using PDS suture and the subcutaneous tissue and skin at the site of the robotic trocars were closed with 2–0 Monocryl and 4–0 Monocryl suture, respectively. Cisplatin 100 mg/m^2^ was infused intraabdominally at 41°C, with concomitant infusion of sodium thiosulfate. The abdomen was irrigated with 6 L Lactated Ringers once HIPEC was completed. The HIPEC apparatus was removed and noted to be intact. The 4.5 cm supraumbilical fascial incision was closed with 0-vicryl. The subcutaneous tissue of all incisions was reapproximated with 2–0 Monocryl and skin reapproximated with 4–0 Monocryl. The case duration including HIPEC was 5 h and 24 min. The patient received the remainder of her sodium thiosulfate infusion per protocol and was admitted to a step-down unit for monitoring after HIPEC. The patient’s recovery was uncomplicated, and she was discharged home on postoperative day two. She resumed systemic chemotherapy 4 weeks after surgery and had no treatment delays.

## Discussion

Advanced stage EOC is associated with 5-year survival of 17%–36% [7]. The addition of HIPEC for stage III disease is associated with improvements in both PFS and OS. Additionally, HIPEC at the time of CRS has been found to be cost effective with significant increases in life years saved [3]. HIPEC has traditionally been performed with laparotomy and that is associated with its own surgical risks, especially in obese patients. Laparotomy in morbidly obese patients is associated with increased length of surgery, and increased rate of complications including surgical site infection, venous thromboembolism, and renal complications [8, 9]. Compared to open surgery, minimally invasive surgery has been demonstrated to have fewer complications including surgical site infections in obese patients, across multiple surgical specialties [10, 11]. This is especially important in gynecologic oncology, where postoperative complications may result in delays in the administration of adjuvant chemotherapy. Historically, an open approach is used for CRS. However, small retrospective studies have demonstrated safety and similar oncologic outcomes with MIS for patients with small volume disease [12, 13].

While the combination of HIPEC and MIS should be associated with decreased morbidity and mortality, laparoscopic HIPEC is associated with additional costs, specifically equipment costs, and perfusionist training, which would result in increased costs for patients. Our case demonstrates the successful pairing of MIS and HIPEC without incurring additional costs. We were able to proceed with MIS CRS and subsequently used standard HIPEC devices as opposed to laparoscopic HIPEC apparatuses. Using standard HIPEC devices avoids costs associated with purchasing new laparoscopic specific equipment, and training perfusionists and other operating room staff on proper use. Recent literature has illustrated high levels of financial toxicity among Gynecologic Oncology patients undergoing cancer treatment [14]. This further emphasizes the importance of finding lower cost solutions while still making advances in the field. Our case report demonstrates a safe and cost-effective approach to MIS CRS and HIPEC in a high risk patient.

## Conclusion

Performing minimally invasive CRS and HIPEC reduced this patient’s hospitalization to 2 days, compared to 4–5-day hospitalization that is typical for open CRS and HIPEC cases. Additionally, the standard laparotomy HIPEC apparatus was used, as opposed to laparoscopic-HIPEC equipment which allowed us to reduce the physical complication rate and the financial burden on this patient. The outcomes of this case, in addition to small case series supporting the safety and efficacy of laparoscopic CRS and HIPEC, suggests that there may be benefit in further investigation into this method of CRS and HIPEC.
